# Children’s Exposure to OP Pesticides: Response to Fenske et al.

**Published:** 2004-11

**Authors:** Gloria D. Coronado, Beti Thompson, William C. Griffith

**Affiliations:** Cancer Prevention Research Program, Fred Hutchinson Cancer Research Center, Seattle, Washington, E-mail: gcoronad@fhcrc.org; Department of Environmental and Occupational Health Sciences, School of Public Health and Community Medicine, University of Washington, Seattle, Washington

In our article (Coronado et al. 2004), we reported a higher proportion of urine samples containing detectable levels of the organophosphate (OP) pesticide urinary metabolite dimethylthiophosphate (DMTP) from children of farmworkers who reported having thinned plants, compared with urine samples from children of non-thinners. We reported the detection frequency for individual dimethyl metabolites, not a composite score for the detection of multiple dimethyl metabolites. We thank Fenske et al. for their additional analyses showing slightly higher, though not significant, concentrations of urinary DMTP in children of thinners versus non-thinners.

We knew that assessing detection frequencies would provide only a preliminary view of a more complex pattern of exposure; thus, we specifically stated in the “Methods” section of our paper that the analysis was exploratory in nature. We examined job task as a factor possibly associated with high exposure to pesticides because job task is closely linked with regulatory policy. We understood that if substantial differences in the percentage of detectable samples existed between groups further exploration would be warranted.

This type of analysis follows the logic put forth by others in the field of exposure assessment. For example, Fenske et al. highlight that Barr et al. (2004)—in the same issue of *EHP* in which our article was published—provided detection frequencies of OP pesticide urinary metabolites in older children (6–11 years of age) from the general population. Barr et al. reported a detection frequency for DMTP of 69%, with a limit of detection of 0.18 μg/L. In our study we matched children (2–6 years of age) with an adult agricultural worker in the same home. Among the children matched to farmworkers who reported thinning, we observed a detection frequency for urinary DMTP of 92%, with a limit of detection of 1.1 μg/L.

We agree with Fenske et al. that a more in-depth analysis is warranted and thank them for their interest and recommendations.

## References

[b1-ehp0112-a0866a] Coronado GD, Thompson B, Strong L, Griffith WC, Islas I (2004). Agricultural task and exposure to organophosphate pesticides among farmworkers. Environ Health Perspect.

[b2-ehp0112-a0866a] Barr DB, Bravo R, Weerasekera G, Caltabiano LM, Whitehead RD, Olsson AO (2004). Concentrations of dialkyl phosphate metabolites of organophosphorus pesticides in the U.S. population. Environ Health Perspect.

